# 2312. COVID-19 risk factors before and after the vaccines among hospital workers: Have they changed?

**DOI:** 10.1093/ofid/ofad500.1934

**Published:** 2023-11-27

**Authors:** Fernando Rosso, David E Rebellon-Sanchez, Julio Llanos-Torres, Eric Tafurt

**Affiliations:** Fundación Valle del Lili, Cali, Valle del Cauca, Colombia; Fundacion Valle del Lili, Cali, Valle del Cauca, Colombia; Fundación Valle del Lili, Cali, Valle del Cauca, Colombia; Fundación Valle del Lili, Cali, Valle del Cauca, Colombia

## Abstract

**Background:**

COVID-19 has been a major public health concern globally, and risk factors have played a crucial role in determining the outcomes of the disease. With the introduction of vaccines, the impact of these risk factors could change and alter the course of the pandemic.

**Methods:**

Ambispective observational study performed among hospital workers of a university hospital in Cali, Colombia. From March 6th, 2020, to February 28th, 2022. Risk factors were assessed with a self-reported survey. Using the aRR calculated for each of the five waves of COVID-19 in Colombia, mixed-effects models were constructed to obtain the association strengths in the pre- and post-vaccination era.

**Results:**

A total of 480 patients, 71.8% were females. The median age was 32 years (27 - 39). In the pre vaccination era there were 169 (35.21%) COVID-19 cases and 165 cases (34.3%) in the post vaccination. In the pre-vaccination era, associated factors were being a healthcare worker (HCW) (aRR 2.12 (95%CI: 1.19-3.79)), technician (aRR 1.73 (1.26-2.38)), living with COVID-19 patients (aRR 2.10 (1.39-3.18)), immunosuppressive medication(aRR 3.88 (1.90-7.70)), self-perceived continuous exposure to COVID-19 patients (aRR 0.28 (0.21-0.37)), and a first COVID-19 episode during the first wave (aRR 0.05 (0.01-0.35)). In the post-vaccination era, COVID-19 history (aRR 0.49 (0.34-0.70)), vaccination in the past 6 months (aRR 0.13 (0.07-0.24)), positive anti-S antibodies in the last 3 months (aRR 0.55 (0.31-0.97)), and positive anti-N antibodies in the last 6 months (aRR 0.35 (0.17-0.74)) were associated.

**Figure 1.**
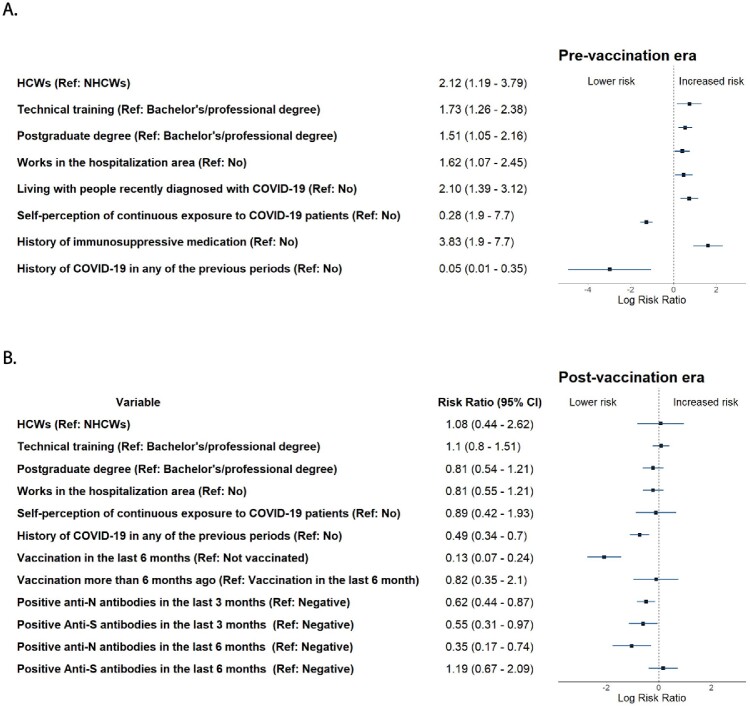
Risks factors for COVID-19 in pre and post vaccination era.

In the pre-vaccination era, being a healthcare worker (HCW), technician, living with COVID-19 patients, immunosuppressive medication, self-perceived continuous exposure to COVID-19 patients and a first COVID-19 episode during the first wave were risk factors. while in the post-vaccination era, COVID-19 history, vaccination in the past 6 months positive anti-S antibodies in the last 3 months, and positive anti-N antibodies in the last 6 months were associated with COVID-19.

**Conclusion:**

This study highlights the dynamic nature of risk factors after the introduction of vaccination. Educational level, being a healthcare worker, working in hospitalization areas, and living with COVID-19 patients were significant risk factors before vaccination. While increased positivity and S-antibody levels linked to vaccination were associated to lower risk of COVID-19 in the post-vaccination era.

**Disclosures:**

**All Authors**: No reported disclosures

